# Remote Sensing Data with the Conditional Latin Hypercube Sampling and Geostatistical Approach to Delineate Landscape Changes Induced by Large Chronological Physical Disturbances

**DOI:** 10.3390/s90100148

**Published:** 2008-01-07

**Authors:** Yu-Pin Lin, Hone-Jay Chu, Cheng-Long Wang, Hsiao-Hsuan Yu, Yung-Chieh Wang

**Affiliations:** Department of Bioenvironmental Systems Engineering, National Taiwan University, 1, Sec. 4, Roosevelt Rd., Da-an District, Taipei City 106, Taiwan, R. O. C; E-Mails: honejaychu@gmail.com (H.-J. C.); r96622053@ntu.edu.tw (C.-L. W.); b94602038@ntu.edu.tw (H.-H. Y.); b92602015@ntu.edu.tw (Y.-C. W.)

**Keywords:** Chi-Chi earthquake, typhoons, landscape changes, remotely sensed images, geostatistics, spatial patterns, Latin hypercube sampling, conditional simulation

## Abstract

This study applies variogram analyses of normalized difference vegetation index (NDVI) images derived from SPOT HRV images obtained before and after the ChiChi earthquake in the Chenyulan watershed, Taiwan, as well as images after four large typhoons, to delineate the spatial patterns, spatial structures and spatial variability of landscapes caused by these large disturbances. The conditional Latin hypercube sampling approach was applied to select samples from multiple NDVI images. Kriging and sequential Gaussian simulation with sufficient samples were then used to generate maps of NDVI images. The variography of NDVI image results demonstrate that spatial patterns of disturbed landscapes were successfully delineated by variogram analysis in study areas. The high-magnitude Chi-Chi earthquake created spatial landscape variations in the study area. After the earthquake, the cumulative impacts of typhoons on landscape patterns depended on the magnitudes and paths of typhoons, but were not always evident in the spatiotemporal variability of landscapes in the study area. The statistics and spatial structures of multiple NDVI images were captured by 3,000 samples from 62,500 grids in the NDVI images. Kriging and sequential Gaussian simulation with the 3,000 samples effectively reproduced spatial patterns of NDVI images. However, the proposed approach, which integrates the conditional Latin hypercube sampling approach, variogram, kriging and sequential Gaussian simulation in remotely sensed images, efficiently monitors, samples and maps the effects of large chronological disturbances on spatial characteristics of landscape changes including spatial variability and heterogeneity.

## 1. Introduction

The influences of large physical disturbances on ecosystem structure and function have garnered considerable attention [1-4]. Fires, hurricanes (typhoons), tornados, ice storms, and landslides are examples of such large disturbances [4]. Earthquakes have long been recognized as a major cause of landslides [5, 6]. However, landslides are only the first in a series of processes by which materials are removed from slopes and transported out of a region by fluvial action [6, 7]. Additionally, typhoons are extremely important natural disturbances that characterize the structure, function and dynamics of many tropical and temperate forest ecosystems [8]. Taiwan, which is located in a subtropical region, sits on the Philippine plate at the Euro-Asian Plate junction [9]. Plate convergence occasionally generates earthquakes that have disastrous effects on Taiwan [10]. Moreover, typhoons that bring tremendous amounts of rainfall hit Taiwan every year from July to October [11]. During 1996–2004, large disturbances in the following sequence impacted central Taiwan: (1) typhoon Herb (August 1996); (2) the Chi-Chi earthquake (September 1999); (3) typhoon Xangsane (November 2000); (4) typhoon Toraji (July 2001); (4) typhoon Dujuan (September, 2003); and, (5) typhoon Mindulle (June 2004) [11]. In particular, after the ChiChi earthquake, the expansion rate of landslide areas increased 20-fold in central Taiwan [12]. Numerous extension cracks, which accelerate landslides during downpours, were generated on hill slopes during the ChiChi earthquake [13]. Moreover, during typhoon seasons, a massive amount of loose earth and stones accumulated on the surface of slopes, increasing the risk of debris flows and additional landslides [14] that worsen the revegetation problem. Accordingly, monitoring, delineating and sampling landscape changes, spatial structure and spatial variation induced by large physical disturbances are essential to landscape management and restoration, and disaster management in Taiwan.

Remotely sensed data can describe surface processes, including landscape dynamics, as such data provide frequent spatial estimates of key earth surface variables [15, 16]. For example, the SPOT, LANSAT and MODIS data sets have notable advantages that account for their use in ecological applications, including a long-running historical time-series, a special resolution appropriate to regional land-cover and land-use change investigations, and a spectral coverage appropriate to studies of vegetation properties [17-19]. The Normalized Difference Vegetation Index (NDVI), a widely used vegetation index, is typically used to quantify landscape dynamics, including vegetation cover and landslides changes induced by large disturbances [6, 8, 11, 16, 20]. Notably, NDVI images can be determined by simply geometric operations near-infrared and visible-red spectral data almost immediately after remotely sensed data is obtained. The NDVI, which is the most common vegetation index, has been extensively used to determine the vigor of plants as a surrogate measure of canopy density [21]. A high NDVI indicates a high level of photosynthetic activity [22]. Moreover, significant differences in NDVI images before and after a natural disturbance can represent landscape changes, including vegetation and landslides induced by a disturbance that changes plant-covered land to bare lands or bare lands to plant-covered land [23].

Spatial patterns in ecological systems are the result of an interaction among dynamic processes operating across abroad range of spatial and temporal scales [24-26]. Ecological manifestations of large disturbances are rarely homogeneous in their spatial coverage [4]. Variograms are crucial to geostatistics. A variogram is a function related to the variance to spatial separation and provides a concise description of the scale and pattern of spatial variability [27]. Samples of remotely sensed data (e.g., satellite or air-borne sensor imagery) can be employed to construct variograms for remotely sensed research [27]. Moreover, variograms have been used widely to understand the nature and causes of spatial variation within an image [28]. Modeling the variogram of NDVI images with high spatial resolution is an efficient approach for characterizing and quantifying heterogeneous spatial components (spatial variability and spatial structure) of a landscape and the spatial heterogeneity of vegetation cover at the landscape level [28, 29].

Reliable data analysis of spatially distributed data requires the use of appropriate statistical tools and a sound data sampling strategy [30]. Spatial sampling schemes have been developed to determine the sampling locations that cover the variation in environmental properties in a given area [31]. Moreover, data samples are transformed via a series of interpretation steps to obtain complete descriptions of phenomena of interest [32]. Different sampling schemes are, say, random, systematic, stratified, or nested schemes [32, 33]. Latin hypercube sampling (LHS) is a stratified random procedure that is an efficient way of sampling variables from their multivariate distributions [34]. Initially developed for Monte-Carlo simulation, LHS efficiently selects input variables for computer models [35, 36]. Kriging, a geostatistical method, is a linear interpolation approach that provides a best linear unbiased estimator (BLUE) for quantities that vary spatially [37]. However, kriging interpolate algorithms generate maps of best local estimate and generally smooth out the local details of the spatial variation of an attribute [38].For sampled data, a geostatistical conditional simulation technique, such as sequential Gaussian simulation (SGS), can be applied to generate multiple realizations, including an error component, which is absent from classical interpolation approaches [37]. In such conditional simulations, all generated realizations reproduce available data at measurement locations, and, on average, reproduce a data histogram and a model of spatial correlations (i.e., variogram) between observations [39]. In SGS, Gaussian transformation of available measurements is simulated, such that each simulated value is conditional on original data and all previously simulated values [37, 40]. Geostatistical conditional simulations have been widely applied to simulate the spatial variability and spatial distribution of interest in many fields. Moreover, geostatistical simulation techniques with LHS have been applied to simulate Gaussian random fields [39, 41-43].

This study applied variogram analysis to delineate spatial variations of NDVI images before and after large physical disturbances in central Taiwan. The NDVI data derived from SPOT images before and after the ChiChi earthquake (ML=7.3 on the Richter scale) in the Chenyulan basin, Taiwan, as well as images before and after four large typhoons (Xangsane, Toraji, Dujuan and Mindulle) were analyzed to identify the spatial patterns of landscapes caused by these major disturbances. Landscape spatial patterns of different disturbance regimes were discussed. Moreover, conditional LHS (cLHS) schemes with NDVI images were used to select spatial samples from actual NDVI images to detect landscape changes induced by a series of large disturbances. The best cLHS samples selected with the NDVI values were used to estimate and simulate NDVI distributions using kriging and SGS. The simulated NDVI images were compared with actual NDVI images induced by the disturbances.

## 2. Methods and Materials

### 2.1. Study area and remote sensing data

**Figure 1.**
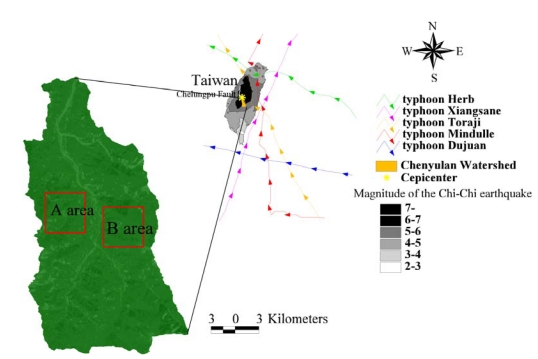
Location of the study areas.

The Chenyulan watershed, located in central Taiwan, is a classical intermountain watershed, and has an average altitude of 1,540 m and an area of 449 km^2^ (Figure 1). The Chenyulan stream, which coincides with the Chenyulan fault, flows from south to north and elongates the watershed in the same direction. Differences in uplifting along the fault generated abundant fractures over the watershed and resulted in an average slope of 62.5% and relief of 585 m/km^2^. Moreover, the main course of the Chenyulan stream had a gradient of 6.1%, and more than 60% of its tributaries had gradients exceeding 20%. The special geological and geographical characteristics of the watershed result in frequent landsides and debris flows [12]. The September 21, 1999, Chi-Chi earthquake occurred at 1:47 a.m. local time (17:47:18 GMT the previous day) at an epicentral location of 23.85_N and 120.78_E and at a depth 6.99 km (Figure 1). It was caused by a rupture in the Chelungpu Fault. The magnitude of the earthquake was estimated to be ML = 7.3 (ML: Local Magnitude or Richter Magnitude), and the rupture zone, defined by the aftershocks, measured about 80 km north-south by 25–30 km downdip [10, 44]. Iso-contour maps of the earthquake's magnitude were reproduced from the Central Weather Bureau (Figure 1) [45]. After the earthquake, from October 31, 2000 to November 1, 2000, the center of typhoon Xiangsane moved from south to north through eastern Taiwan [46], with a maximum wind speed of 138.9 km/hr and a radius of 250 km (Figure 1). The maximum daily rainfall was 550 mm/day. On July 30, 2001, the Toraji typhoon swept across central Taiwan from east to west [47], with a maximum wind speed of 138.9 km/hr and a radius of 180 km (Figure 1). The typhoon brought extremely heavy rainfall, from 230 to 650 mm/ day, and triggered more than 6000 landslides in Taiwan. After crossing Taiwan, typhoon Toraji became a tropical storm; however it brought 339 to 757 mm of total accumulated rainfall in the watershed [47] (Figure 1). After typhoon Toraji, typhoons Dujuan with a maximum wind speed of 165.0 km/hr, a radius of 200 km and maximum rainfall 200 mm/hr (August 31, 2003–September 2, 2003) and Mindulle with maximum wind speed of 200.0 km/hr, a radius of 200 km and maximum rainfall 166 mm/hr (June 29, 2004–July 2, 2004) chronologically produced heavy rainfall that fell across the eastern and central parts of Taiwan on September 2003 and June 2004 [48] (Figure 1). The two study area with dimensions of 50×50 km^2^ (250×250 pixels) was selected from the upstream of the large debris flood announced in the watershed, as shown in Figure 1.

### 2.2. NDVI

Seven cloud-free SPOT images (1996/11/08, 1999/03/06, 1999/10/31, 2000/11/27, 2001/11/20, 2003/12/17 and 2004/11/19) of the Chenyulan watershed were purchased from the Space and Remote-sensing Research Center, Taiwan. The NDVI images of the study area were generated from SPOT HRV images with a resolution of 20 m according to the following equation:

(1) $$\text{NDVI}=\frac{\text{NIR}-R}{\text{NIR}+R}$$

where NIR and R are near-infrared and visible-red spectral data, respectively. The NDVI values range from −1 to +1; a high NDVI value represents a large amount of high photosynthesizing vegetation [49].

### 2.3. Variogram and kriging estimation

In geostatistical methods, variograms can be used to quantify the observed relationship between the values of samples and the proximity of samples [37]. Following the work of Garrigues *et al.* (2006), Garrigues *et al.* (2008) and Lin *et al.* (2008), NDVI data are considered values of punctual regionalized variable. An experimental variogram for interval lag distance class *h*, *γ*(*h*), is represented by

(2) $$\gamma (h)=\frac{1}{2n(h)}\sum_{i=1}^{n(h)}{\left[Z(x_{i}+h)-Z(x_{i})\right]}^{2}$$

where *h* is the lag distance that separates pairs of points; *Z(x)* is bird diversity at location x, and *Z(x* + *h*) is bird diversity at location *x* + *h*; *n(h)* is the number of pairs separated by lag distance *h*.

Kriging is estimated using weighted sums of adjacent sampled concentrations. The weights depend on the correlation structure exhibited. The weights are determined by minimizing estimated variance. In this context, kriging estimates (Best Linear Unbiased Estimator) are the most accurate of all linear estimators. Accordingly, kriging estimates the value of the random variable at unsampled location X0based on measured values in a linear form:

(3) $$Z^{*}(x_{0})=\sum_{i=1}^{N}\lambda_{i0}Z(x_{i})$$

where Z* (*x*_0_) is the estimated value at location *x*_0_, *λ*_i0_ is the estimation weight of *Z*(*x_i_*), *x_i_* is the location of sampling point for variable Z, and N is the number of the variable Z involved in the estimation.

Based on non-biased constraints and minimizing estimation variance, estimated kriging variance can be presented as:

(4) $${\sigma^{2}}_{\text{kriging}}=\sum_{i=1}^{N}\lambda_{i0}\gamma_{zz}(x_{i}-x_{0})+\mu$$

where *μ* is the Lagrange multiplier.

### 2.4. Conditional Latin hypercube

The cLHS, which is based on the empirical distribution of original data, provides a full coverage of range each variable by maximally stratifying the marginal distribution and ensuring a good spread of sampling points [34]. This sampling procedure represents an optimization problem: given N sites with ancillary data (*Z*), select n sample sites (*n*≪*N*) such that the sampled sites z form a Latin hypercube. For continuous variables, each component of X (size, *N*×*k*) is divided into n (sample size) equally probable strata based on their distributions, and x (size *n*×*k*) is a sub-sample of X. The procedures of the cLHS algorithm [34] are follows.

1. Divide the quantile distribution of X into n strata, and calculate the quantile distribution for each variable, $q_{j}^{i},\ldots ,q_{j}^{n+1}$. Calculate the correlation matrix for *Z* (C).
2. Pick n random samples from *N*; z (*i*=1,…, *n*) are the sampled sites. Calculate the correlation matrix of x (T).
3. Calculate the objective function. The overall objective function is *O* = *w*_1_*O*_1_ + *w*_2_*O*_2_ + *w*_3_*O*_3_, , where w is the weight given to each component of the objective function. For general applications, w is set to 1 for all components of the objective function.
  - For continuous variables,
    (5) $$O_{1}=\sum_{i=1}^{n}\sum_{j=1}^{k}\left|\eta (q_{j}^{i}\leq x_{j}\leq q_{j}^{i+1})\right|-1$$
    where $\eta (q_{j}^{i}\leq x_{j}\leq q_{j}^{i+1})$ is the number of *x_i_* that falls between quantiles $q_{j}^{i}$ and $q_{j}^{i+1}$
  - For categorical data, the objective function is to match the probability distribution for each class of:
    (6) $$O_{2}=\sum_{j=1}^{C}\left|\frac{\eta \prime (z_{j})}{n}-k_{j}\right|$$
    where *η'*(*x_i_*) is the number of x that belongs to class j in sampled data, and *k_i_* is the proportion of class j in X.
  - C. To ensure that the correlation of the sampled variables will replicate the original data, another objective function is added:
    (7) $$O_{3}=\sum_{i=1}^{k}\sum_{j=1}^{k}\left|c_{ij}-t_{ij}\right|$$
    where c is the element of C, the correlation matrix of X, and t is the equivalent element of T, the correlation matrix of x.
4. Perform an annealing schedule [50]: *M* = exp[-ΔO/T], where ΔO is the change in the objective function, and T is a cooling temperature (between 0 and 1), which is decreased by a factor d during each iteration.
5. Generate a uniform random number between 0 and 1. If *rand* < *M*, accept the new values; otherwise, discard changes.
6. Try to perform changes: Generate a uniform random number rand. If *rand* < *P*, pick a sample randomly from x and swap it with a random site from unsampled sites r. Otherwise, remove the sample(s) from x that has the largest $\eta (q_{j}^{i}\leq x_{j}\leq q_{j}^{i+1})$ and replace it with a random site(s) from unsampled sites r. End when the value of *P* is between 0 and 1, indicating that the probability of the search is a random search or systematically replacing the samples that have the worst fit with the strata.
7. Go to step 3
  Repeat steps 3–7 until the objective function value falls beyond a given stop criterion or a specified number of iterations.

### 2.5. Sequential Gaussian Simulation

In sequential simulation algorithm, modeling of the N-point cumulative density function (ccdf) is a sequence of N univariate ccdfs at each node (grid cell) along a random path [39]. The sequential simulation algorithm has the following steps [39]:

1. Establish a random path that is visited once and only once, all nodes {*u_i_, i* = 1, Λ, N} discretizing the domain of interest Doman. A random visiting sequence ensures that no spatial continuity artifact is introduced into the simulation by a specific path visiting N nodes.
2. At the first visited N nodes *u*_1_:
  - Model, using either a parametric or nonparametric approach, the local ccdf of *Z*(*u*_1_) conditional on n original data {*Z* (*u_α_*), *α* = 1,Λ, *n*} *F_Z_* (*u*_1_; *z*_1_|(*n*)) = *prob* {*Z* (*u*_1_) ≤ *z*_1_|(*n*)}
  - Generate, via the Monte Carlo drawing relation, a simulated value *z*^(^*^l^*^)^(*u*_1_) from this ccdf *F_Z_* (*u*_1_: *z*_1_|(*n*)), and add it to the conditioning data set, now of dimension *n* + 1, to be used for all subsequent local ccdf determinations.
3. At the i_th_ node *u_i_* along the random path:
  - Model the local ccdf of *Z*(*u_i_*) conditional on n original data and the *i* - 1 near previously simulated values { *z*^(^*^l^*^)^(*u_i_*), *j* = 1,Λ, *i* - 1}:
    (8) $$F_{Z}(u_{1};z_{i}|\left(n+i-1\right))=\text{prob}\{Z(u_{i})\leq z_{i}|\left(n+i-1)\right\}$$
  - Generate a simulated value *z*^(^*^l^*^)^(*u_i_*) from this ccdf and add it to the conditioning data set, now of dimension *n* + *i*.
4. Repeat step 3 until all N nodes along the random path are visited.

The SGS assumes a Gaussian random field, such that the mean value and covariance completely characterize the ccdf [51]. During the SGS process, Gaussian transformation of available measurements is simulated, such that each simulated value is conditional on original data and all previously simulated values [14, 40]. A value simulated at a one location is randomly selected from the normal distribution function defined by the kriging mean and variance based on neighborhood values. Finally, simulated normal values are back-transformed into simulated values to yield the original variable. The simulated value at the new randomly visited point value depends on both original data and previously simulated values. This process is repeated until all points have been simulated.

## 3. Results and Discussion

### 3.1. Statistics and spatial structures of NDVI images

**Figure 2.**
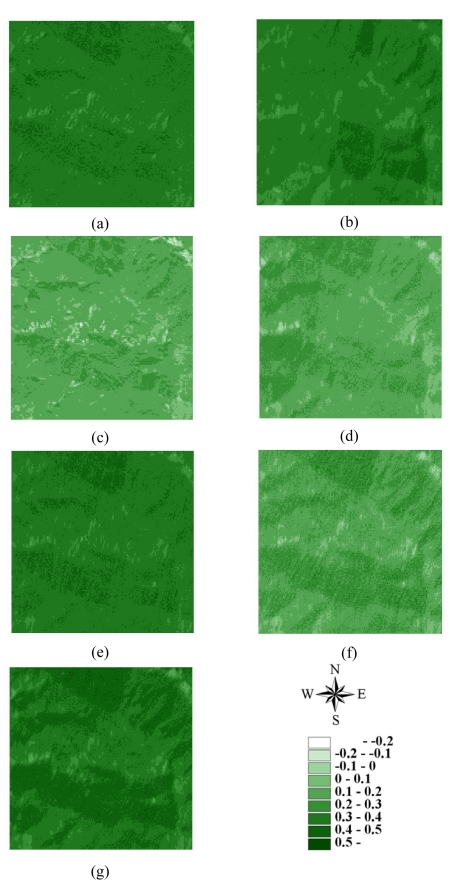
NDVI images of area A on (a) 1996/11/08, (b) 1999/03/06, (c) 1999/10/31, (d) 2000/11/27, (e) 2001/11/20, (f) 2003/12/17, and (g) 2004/11/19.

**Figure 3.**
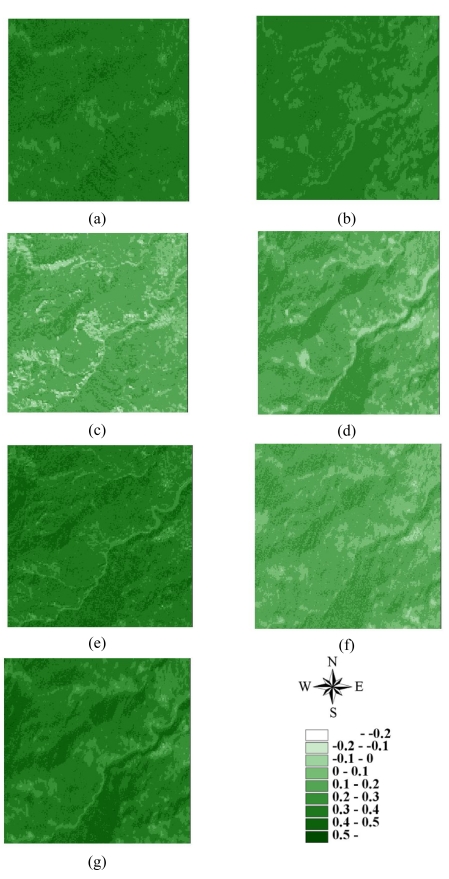
NDVI images of area B on (a) 1996/11/08, (b) 1999/03/06, (c) 1999/10/31, (d) 2000/11/27, (e) 2001/11/20, (f) 2003/12/17, and (g) 2004/11/19.

Statistics of remotely sensed images can be used as a basic tool to characterize landscape changes [52-56]. Table 1 summaries the statistics for seven actual NDVI images of areas A and B before and after disturbances. The lowest mean and minimum NDVI values in 1996–2004 occurred on March 6, 1999, after the Chi-Chi earthquake in both areas A and B areas. Moreover, the largest range between minimum and maximum NDVI values also occurred on March 6, 1999, after the Chi-Chi earthquake in both areas A and B. The most negative minimum NDVI values occurred on November 27, 2000, and December 17, 2003, in both areas A and B. On these dates, the standard deviations of NDVI values were slightly larger than those on other dates. These statistical results illustrate that the Chi-Chi earthquake had the largest impact on all landscapes represented by NDVI images for areas A and B. The second and third greatest impacts on all landscapes are from typhoons Xangsane (November 2000) and Dujuan (September 2003) in areas A and B, respectively (Figures 2 and 3 and Table 1). Particularly, typhoon Xangsane right after the ChiChi earthquake was the second disturbance to impact landscape changes in the study areas. Numerous extension cracks, which increase the number of landslides during downpours, were generated on hill slopes during the Chi-Chi earthquake [13]. Statistical results illustrate that the effects of disturbances on the watershed landscape in the study areas were cumulative, but were not always evident in space and time over the entire landscape [13]. The effects of the Chi-Chi earthquake on the landscapes of the study areas gradually declined; this finding was also obtained by Chang *et al.* (2007). However, in the Chenyulan watershed, as the landslide ratio increased with successive rainstorms and strong earthquakes, the NDVI values decreased [11]. Hence, subsequent rainstorms cause divergent destruction of vegetation; this destruction may be influenced by the precipitation distribution and typhoon path [11] (Table 1).

**Figure 4.**
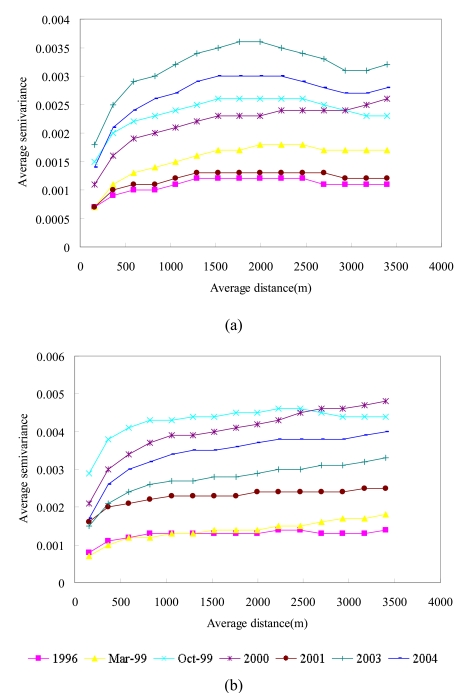
Experimental variograms of NDVI images before and after disturbances in areas (a) A and (b) B.

Previous studies that quantified the impact of large disturbances did not evaluate the spatial structures of NDVI images in the study areas. To demonstrate the ability of the variogram to depict landscape heterogeneity, spatial variability and patterns, experimental variograms and their variogram models were first analyzed and fit to seven images of areas A and B (Figure 4 and Table 2). The models are obtained in two processes such as parameter estimation (fitting) and cross validation. Cross-validation in Table 2 is a means for evaluating effective parameters for kriging interpolations. In cross-validation analysis each measured point in a spatial domain is individually removed from the domain and its value estimated via kriging as though it were never there. In this way a graph can be constructed of the estimated vs. actual values for each sample location in the domain.

The three main features of a typical variogram model are (1) the range, (2) the sill, and (3) the nugget effect. The sill is the upper limit that a variogram approaches at a large distance, and is a measure of the variability of the investigated variable: a higher sill corresponds to greater variability in the variable. The range of a variogram model is the distance lag at which the variogram approaches the sill, and can reveal the distance above which the variables become spatially independent. The nugget effect is exhibited by the apparent non-zero value of the variogram at the origin, which may be due to the small-scale variability of the investigated process and/or measured errors. In this study, the variogram models of the seven NDVI images for areas A and B areas are exponential models. The spatial variations (Sill; *C*_0_ + *C*) of NDVI images from high to low are in 2003/12/17, 2004/11/19, 1999/10, 2000/11/27, 1999/03/06, 2001/11/20 and 1996/11/08 in area A. The spatial variations (Sill; *C*_0_ + *C*) of NDVI images from high to low in area B are in 1999/10/31, 2000/11/27, 2004/11/19, 2003/12/17, 2001/11/20, 1999/03/06 and 1996/11/08. The spatial variations of NDVI images increase considerably from 1996/11/08 to 1999/10/31 (after the Chi-Chi earthquake) in both areas A and B. Similarly, small variations (Nugget effect) of NDVI images in 2003/12/17 (after typhoon Dujuan), 1999/10 (after the Chi-Chi earthquake) and 2000/11/27 in area A are larger than those in 1999/03/06, 2001/11/20, 1996/11/08 and 2004/11/19. In area B, small variations (Nugget effects) of NDVI images in 1999/10/31 are larger than those in other images. As the range of a variogram model increases, the continuity of an NDVI image increases. The ranges of NDVI variogram models in area A from long range to short range are in 2000/11/27, 2001/11/20, 1999/03/06, 1996/11/08, 1999/10, 2003/12/17, and 2004/11/19. In area B, the ranges of NDVI variogram models from long range to short range are in 1999/03/06, 2000/11/27, 2003/12/17, 2004/11/19, 2001/11/20, 1996/11/08, and 1999/10. However, exponential models with large sills, large nugget effects and short-range NDVI images are indicative of significant spatial heterogeneous landscapes induced by the Chi-Chi earthquake in areas A and B. Moreover, typhoons Xangsane and Dujuan generated heterogeneous landscapes in area A.

High-spatial-resolution observations (e.g., SPOT-HRV, pixel size of 20 m) capture most landscape spatial heterogeneity and are thus can be used to quantify the spatial heterogeneity within moderate spatial resolution pixels [16, 29]. The shape of variograms can be used to understand the NDVI spatial structures within an image domain [29]. Millward and Kraft (2004) applied variograms to evaluate the impacts of disturbances on landscapes. In this study, experimental variogram and modeling results indicate that large disturbances, such as the Chi-Chi earthquake, created extremely complex heterogeneous patterns across the landscape. Notably, a disturbance may affect some areas but not others, and disturbance severity often varies considerably within an affected area on the landscape level [3, 13]. Variography results illustrate that NDVI discontinuities between fields create a mosaic spatial structure resulting primarily from large disturbances, such as the Chi-Chi earthquake, in the study areas. Moreover, the high-magnitude Chi-Chi earthquake created these landscape variations in space in the Chenyulan watershed [13]. Previous studies [11, 13, 57] indicated that landslides in the Chenyulan watershed were impacted by the Chi-Chi earthquake; however, the effect of the earthquake decreased as the time between a typhoon and the Chi-Chi earthquake increased [57]. Moreover, variography results confirm that the impacts of disturbances on the watershed landscape pattern were cumulative, but were not always evident in space and time in the entire landscape [23, 57]. Moreover, landslides induced by earthquakes and typhoons have distinct spatial patterns [11]. Typhoons significantly influence NDVI variations via the flow of accumulated rainfall and wind gradients [37]. The statistical and variogram results also indicate that basic statistics without variograms of NDVI images may not sufficient to present landscape changes induced by disturbances, particularly via spatial structure, variability and heterogeneity analysis. Moreover, variogram modeling results also support the above statistical results, indicating that subsequent rainstorms may cause divergent destruction of vegetation, and then this destruction may be influenced by the precipitation distribution and typhoon path [12, 13].

### 3.2. Latin hypercube sampling for multiple images

**Figure 5.**
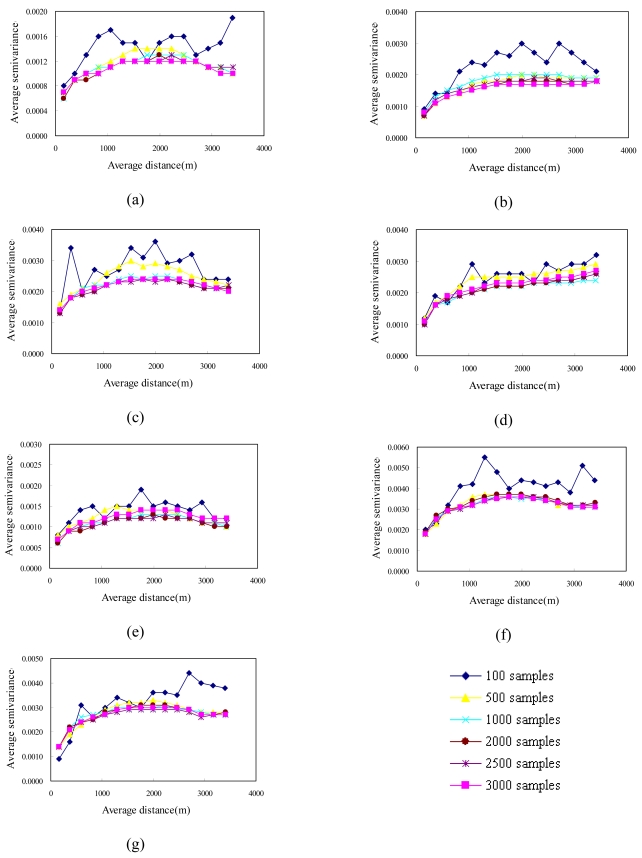
Experimental variograms of NDVI samples for area A on (a) 1996/11/08, (b) 1999/03/06, (c) 1999/10/31, (d) 2000/11/27, (e) 2001/11/20 (f) 2003/12/17, and (g) 2004/11/19.

**Figure 6.**
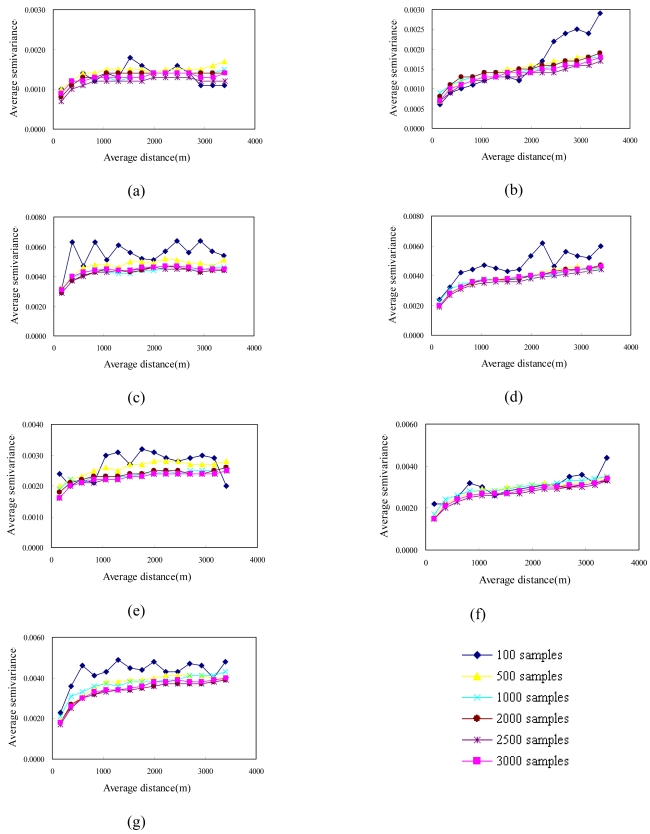
Experimental variograms of NDVI samples for area B on (a) 1996/11/08, (b) 1999/03/06, (c) 1999/10/31, (d) 2000/11/27, (e) 2001/11/20, (f) 2003/12/17, and (g) 2004/11/19.

Sampling strategies are typically based on an assumed theoretical framework (Edwards and Fortin, 2001). Sampling under such a framework involves attempting to locate samples to capture the possible variations and fluctuations in a gradient field [32]. An efficient sampling method is therefore needed to cover the entire range of ancillary variables [34]. In this study, experimental variograms of cLHS samples with their NDVI values were constructed using the same lag interval to compare the spatial structures of the actual NDVI images. Figures 5 and 6 show experimental variograms for 100, 500, 1,000, 2,000, 2,500 and 3,000 cLHS samples in 1996/11/08, 1999/03/06, 1999/10/31, 2000/11/27, 2001/11/20, 2003/12/17 and 2004/11/19, respectively. These experimental variograms show that as the number of samples increased from 100 to 3000, the ability of experimental variograms to capture the spatial structure of actual NDVI images increased. These variography results show that the cLHS approach can simultaneously select samples from multiple NDVI images to capture spatial structures of all NDVI spatial structures.

**Figure 7.**
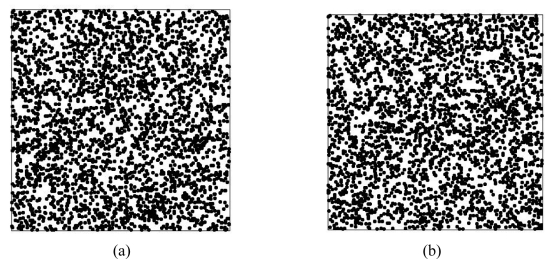
Locations of the 3,000 samples in areas (a) A and (b) B.

Table 3 lists statistics for 100, 500, 1,000 and 3,000 samples from multiple NDVI images with 62,500 grids using the cLHS approach. Figure 7 shows the 3,000 samples selected using cLHS in each area. The distributions of selected samples confirm that samples selected using cLHS provide a good coverage of the study area and are well spread and partially clustered in the study areas [34]. The statistics for these 3,000 samples indicate that the statistics obtained by cLHS can capture statistics of all actual NDVI images. The statistical and variogram analyses of cLHS samples also illustrate that the cLHS approach can be applied to select samples and capture the spatial structures of multiple historically accurate NDVI images. These samples can be used in further monitoring and to determine the impacts of disturbances on study landscapes in the future.

### 3.3. Estimations and conditional simulations with selected samples

**Figure 8.**
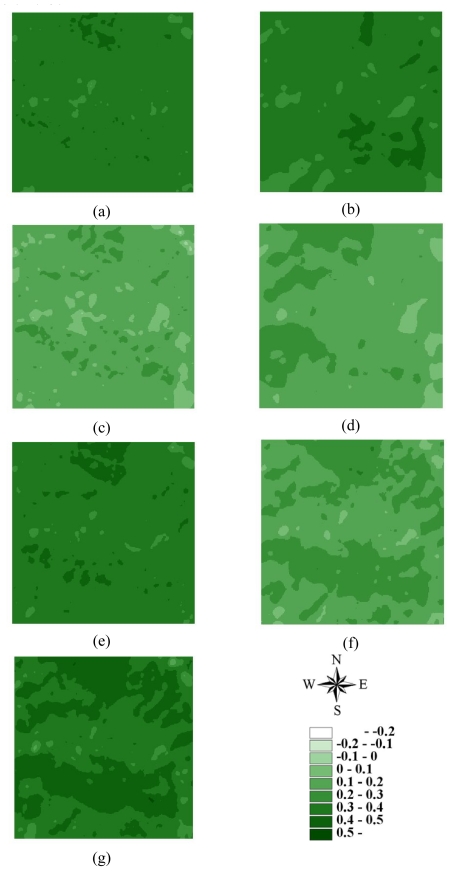
Kriging estimated NDVI images based on 3,000 samples in area A on (a) 1996/11/08, (b) 1999/03/06, (c) 1999/10/31, (d) 2000/11/27, (e) 2001/11/20, (f) 2003/12/17, and (g) 2004/11/19.

**Figure 9.**
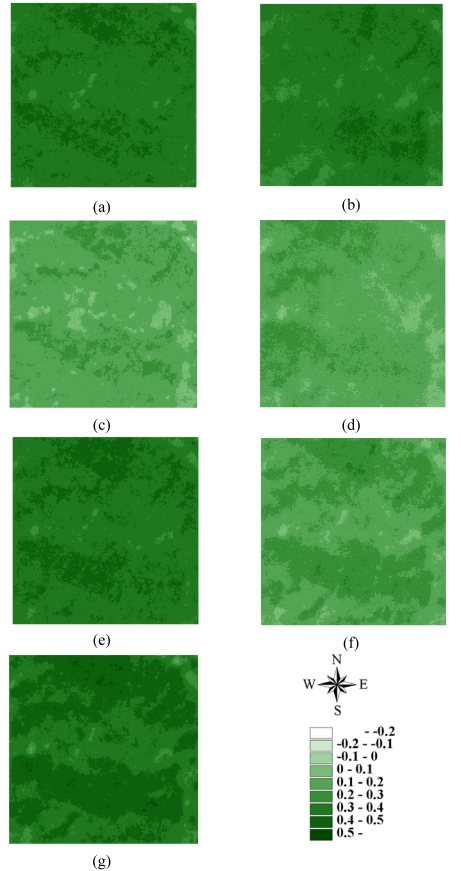
Conditional simulated NDVI images based on 3,000 samples in area A on (a) 1996/11/08, (b) 1999/03/06, (c) 1999/10/31 (d) 2000/11/27, (e) 2001/11/20, (f) 2003/12/17, and (g) 2004/11/19.

**Figure 10.**
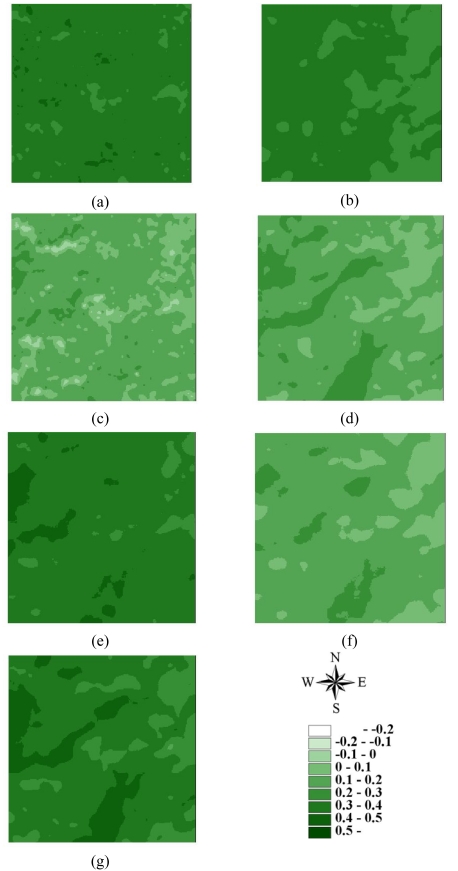
NDVI images estimated by kriging based on 3,000 samples in area B on (a) 1996/11/08, (b) 1999/03/06, (c) 1999/10/31, (d) 2000/11/27, (e) 2001/11/20, (f) 2003/12/17, and (g) 2004/11/19.

**Figure 11.**
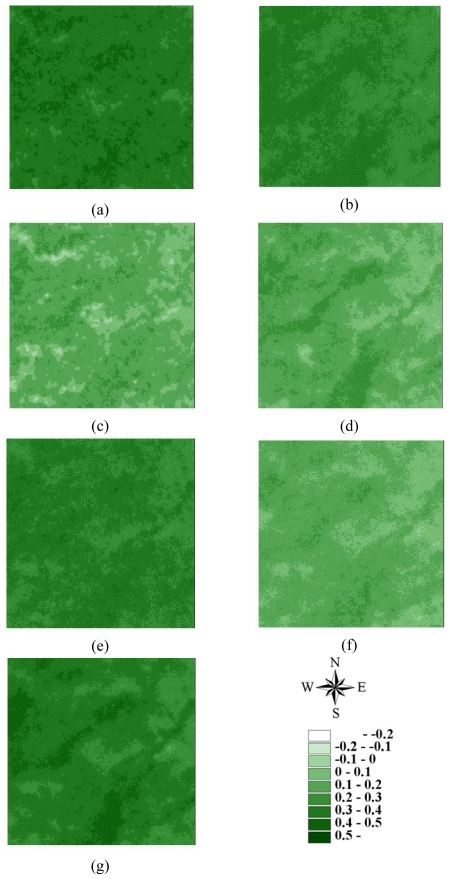
Conditional simulated NDVI images based on 3,000 samples for area B on (a) 1996/11/08, (b) 1999/03/06, (c) 1999/10/31, (d) 2000/11/27, (e) 2001/11/20, (f) 2003/12/17, and (g) 2004/11/19.

**Figure 12.**
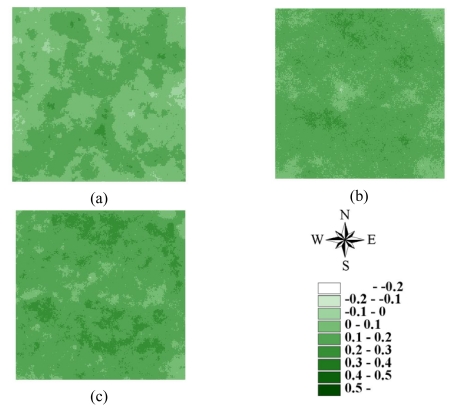
Conditional simulated NDVI images for area A based on (a) 100, (b) 500, and (c) 1,000 cLHS samples on 1999/10/31

**Figure 13.**
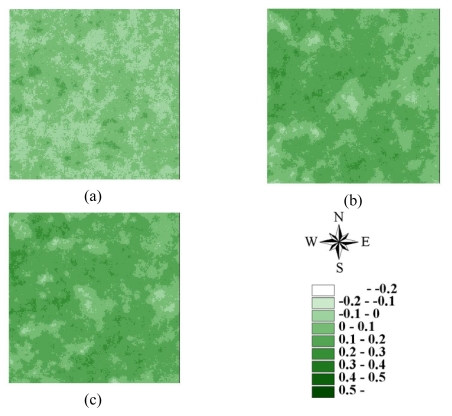
Conditional simulated NDVI images for area B based on (a) 100, (b) 500, and (c) 1,000 cLHS samples on 1999/10/31.

The LHS approach can also be used in SGS [41, 43] and kriging estimation. However, because the LHS is conducted by shifting simple random sampling, meaningful deviations exist when sample size is small [41, 43]. In this study, ordinary kriging estimates and SGS simulations were performed based on the above variogram models of 3000 samples for 7 NDVI images in areas A and B. Figures 8–11 show the maps of kriging and averages of 1000 realizations of SGS of NDVI images in 62500 grids in areas A and B. A comparison of actual NDVI images and NDVI images estimated by kriging indicates that kriging estimation with sufficient samples provides the best local estimates to capture actual NDVI images, but generally smoothed extreme values of the actual NDVI images in areas A and B (Figures 8–11). Figures 12 and 13 show NDVI maps produced by SGS simulations with 100, 500 and 1000 cLHS samples in 1999/10/31 for areas A and B. The kriging estimation results illustrate that interpolation techniques such as kriging typically ignore phase information, which can result in an over-smoothed view of the distribution of spatial variables and remove important information about spatial discontinuities in a pattern [6, 14, 20, 26, 40], particular with an insufficient number of samples (Figures 8-13). However, kriging interpolation algorithms produce maps of the best local estimate and generally smooth local details of spatial variation of an attribute [38].

The SGS results verify that the limits of spatial analysis and interpolations of landscape variables are based on semivariograms (or autocorrelation functions) solely, stressing the need to account for spatial discontinuities [26], particularly in highly heterogeneous landscapes induced by large physical disturbances such as the Chi-Chi earthquake. Therefore, procedures for interpolation of ecological variables must include information on spatial discontinuities, either directly from remotely sensed images (assuming the phase pattern in the image and ecological variables are equivalent) or indirectly by sampling with sufficient intensity spatial variables in the field that have a known functional relationship with the variable of interest (assuming the variable of interest is too difficult or expensive to sample in the field) [26]. The simulated NDVI images show that kriging and SGS and the cLHS approach provide effective tools for monitoring, sampling and mapping landscape changes induced by large disturbances.

## 4. Conclusions

This study presents a novel and effective approach that integrates cLHS, variograms, kriging and SGS in remotely sensed images for efficient monitoring, sampling and mapping of the impacts of chronologically ordered large disturbances on spatial characteristics of landscape changes to spatial structure, variability and heterogeneity. The NDVI images, which can be generated almost immediately after the remotely sensed data are acquired, were used as the inferential index because landscape changes induced by a large disturbance are easily recognized by changes in NDVI images. Variography of multiple NDVI images before and after a large disturbance is an essential method for characterizing and quantifying the spatial variability, structure and heterogeneity of landscapes induced by a disturbance. The variography results illustrated that cumulative impacts of disturbances on spatial variability existed and depended on disturbance magnitudes and paths, but were not always evident in spatiotemporal variability of landscapes in the study areas. Moreover, the cLHS approach is an effective sampling approach for multiple true NDVI images from their multivariate distributions to replicate the statistical distribution and spatial structures of the NDVI images. The sufficient number of NDVI samples using cLHS can be used to monitor and sample changes in landscapes induced by large physical disturbances. Kriging and SGS combined with the sufficient number of cLHS samples can be used to estimate and simulate NDVI images to generate maps that capture the spatial pattern and variability of actual NDVI images of disturbed landscapes. Kriging with sufficient number of NDVI cLHS samples produces NDVI maps with the best local estimates to identify patterns of NDVI images of disturbed landscapes. SGS with sufficient cLHS samples generate multiple realizations and an average of the realizations of NDVI and captures the spatial variability and heterogeneity of disturbed landscapes.

## Figures and Tables

**Table 1.** Statistics of NDVI images.

**Area**	**Date**	**Mean**	**Std.**	**Min.**	**Max.**
**A**	1996/11/08	0.36	0.04	0.11	0.48
	1999/03/06	0.32	0.04	0.13	0.43
	1999/10/31	0.14	0.07	-0.22	0.33
	2000/11/27	0.15	0.07	-0.14	0.35
	2001/11/20	0.37	0.05	0.03	0.50
	2003/12/17	0.15	0.06	-0.12	0.33
	2004/11/19	0.35	0.06	0.05	0.54
	
**B**	1996/11/08	0.36	0.03	0.13	0.47
	1999/03/06	0.36	0.04	0.14	0.48
	1999/10/31	0.16	0.05	-0.20	0.38
	2000/11/27	0.17	0.05	-0.09	0.33
	2001/11/20	0.37	0.04	0.14	0.48
	2003/12/17	0.20	0.06	-0.08	0.44
	2004/11/19	0.39	0.05	0.10	0.57

**Table 2.** Variogram models of NDVI images.

**Area**	**Date**	**Model**	**Parameters**	**The fit**	**Cross-validate**
**A**	1996/11/08	Exponential model	*C*_0_=0.000453, *C*_0_+*C*=0.001212, *R*=1204.000	(SS=7.774E-08; *r*^2^=0.832, *C*_0_/*C*_0_+*C*=0.374)	*r*^2^ =0.722
	1999/03/06	Exponential model	*C*_0_=0.000147, *C*_0_+*C*=0.001744; *R*=1278.000	(SS=3.490E-08; *r*^2^=0.978, *C*_0_/*C*_0_+*C*=0.084)	*r*^2^=0.893
	1999/10/31	Exponential model	*C*_0_=0.000878, *C*_0_+*C*=0.002496; *R*=1020.000	(SS=1.573E-07; *r*^2^=0.873,*C*_0_/*C*_0_+*C*=0.352)	*r*^2^=0.839
	2000/11/27	Exponential model	*C*_0_=0.000761, *C*_0_+*C*=0.002452; *R*=1881.000	(SS=18.597E-08; *r*^2^=0.961, *C*_0_/*C*_0_+C=0.310)	*r*^2^=0.894
	2001/11/20	Exponential model	*C*_0_=0.000518, *C*_0_+*C*=0.001294; *R*=1497.000	(SS=5.124E-08; *r*^2^=0.878, *C*_0_/*C*_0_+*C*=0.400)	*r*^2^=0.723
	2003/12/17	Exponential model	*C*_0_=0.000700, *C*_0_+*C*=0.003370; *R*=981.000	(SS=3.420E-07; *r*^2^=0.893, *C*_0_/*C*_0_+*C*=0.208)	*r*^2^=0.737
	2004/11/19	Exponential model	*C*_0_=0.000229, *C*_0_+*C*=0.002878; *R*=918.000	(SS=1.918E-07; *r*^2^=0.930, *C*_0_/*C*_0_+*C*=0.080)	*r*^2^=0.862
**B**	1996/11/08	Exponential model	*C*_0_=0.000138, *C*_0_+*C*=0.001326; *R*=654.000	(SS=1.610E-08; *r*^2^=0.953, *C*_0_/*C*_0_+C=0.104)	*r*^2^=0.781
	1999/03/06	Exponential model	*C*_0_=0.000712, *C*_0_+*C*=0.001814; *R*=4620.000	(SS=6.070E-08; *r*^2^=0.945, *C*_0_/*C*_0_+*C*=0.393)	*r*^2^=0.901
	1999/10/31	Exponential model	*C*_0_=0.000590, *C*_0_+*C*=0.004440; *R*=564.000	(SS=1.678E-07; *r*^2^=0.939, *C*_0_/*C*_0_+*C*=0.133)	*r*^2^=0.849
	2000/11/27	Exponential model	*C*_0_=0.0001863, *C*_0_+*C*=0.004676; *R*=2646.000	(SS=2.474E-07; *r*^2^=0.952, *C*_0_/*C*_0_+*C*=0.398)	*r*^2^=0.908
	2001/11/20	Exponential model	*C*_0_=0.0001205, *C*_0_+*C*=0.002429; *R*=1281.000	(SS=5.621E-08; *r*^2^=0.933, *C*_0_/*C*_0_+*C*=0.498)	*r*^2^=0.728
	2003/12/17	Exponential model	*C*_0_=0.0001258, *C*_0_+*C*=0.003126; *R*=2298.000	(SS=1.567E-07; *r*^2^=0.949, *C*_0_/*C*_0_+*C*=0.402)	*r*^2^=0.820
	2004/11/19	Exponential model	*C*_0_=0.0001161, *C*_0_+*C*=0.003832; *R*=1680.000	(SS=1.186E-07; *r*^2^=0.977, *C*_0_/*C*_0_+*C*=0.303)	*r*^2^=0.902

*C*_0_=Nugget; *C*_0_+*C*=Sill; *R*= Range

**Table 3.** Statistics of 100, 500, 1,000 and 3,000 samples from NDVI images.

**Area**		**Date**	**Mean**	**Std.**	**Min.**	**Max.**	**Area**		**Date**	**Mean**	**Std.**	**Min.**	**Max.**
**100**	**A**	1996/11/08	0.36	0.04	0.22	0.44	**1,000**	**A**	1996/11/08	0.36	0.03	0.17	0.45
		1999/03/06	0.36	0.05	0.22	0.45			1999/03/06	0.36	0.04	0.17	0.47
		1999/10/31	0.16	0.05	0.00	0.24			1999/10/31	0.16	0.05	-0.10	0.33
		2000/11/27	0.17	0.05	0.00	0.28			2000/11/27	0.17	0.05	0.00	0.33
		2001/11/20	0.37	0.04	0.19	0.44			2001/11/20	0.37	0.04	0.20	0.46
		2003/12/17	0.19	0.06	0.01	0.33			2003/12/17	0.20	0.06	0.00	0.38
		2004/11/19	0.39	0.05	0.19	0.05			2004/11/19	0.40	0.05	0.17	0.54
	**B**	1996/11/08	0.36	0.04	0.24	0.44		**B**	1996/11/08	0.16	0.07	0.00	0.30
		1999/03/06	0.31	0.05	0.20	0.38			1999/03/06	0.36	0.05	0.15	0.47
		1999/10/31	0.13	0.08	-0.08	0.28			1999/10/31	0.15	0.06	-0.04	0.29
		2000/11/27	0.15	0.07	0.00	0.28			2000/11/27	0.36	0.06	0.12	0.50
		2001/11/20	0.35	0.06	0.20	0.46			2001/11/20	0.36	0.04	0.17	0.44
		2003/12/17	0.15	0.07	-0.05	0.29			2003/12/17	0.32	0.04	0.16	0.41
		2004/11/19	0.35	0.08	0.16	0.49			2004/11/19	0.14	0.07	-0.12	0.29
**500**	**A**	1996/11/08	0.37	0.04	0.17	0.44	**3,000**	**A**	1996/11/08	0.36	0.04	0.15	0.46
		1999/03/06	0.36	0.04	0.19	0.46			1999/03/06	0.36	0.04	0.16	0.48
		1999/10/31	0.16	0.05	-0.20	0.26			1999/10/31	0.16	0.05	-0.10	0.30
		2000/11/27	0.17	0.05	0.00	0.31			2000/11/27	0.17	0.05	0.00	0.33
		2001/11/20	0.37	0.04	0.19	0.45			2001/11/20	0.37	0.04	0.15	0.48
		2003/12/17	0.20	0.06	0.00	0.36			2003/12/17	0.20	0.06	0.00	0.44
		2004/11/19	0.40	0.06	0.17	0.53			2004/11/19	0.39	0.06	0.13	0.57
	**B**	1996/11/08	0.35	0.04	0.17	0.44		**B**	1996/11/08	0.36	0.04	0.20	0.46
		1999/03/06	0.32	0.04	0.18	0.40			1999/03/06	0.32	0.04	0.16	0.41
		1999/10/31	0.13	0.07	-0.15	0.25			1999/10/31	0.14	0.07	-0.19	0.33
		2000/11/27	0.15	0.06	0.00	0.30			2000/11/27	0.15	0.07	0.00	0.32
		2001/11/20	0.36	0.05	0.17	0.46			2001/11/20	0.36	0.05	0.07	0.47
		2003/12/17	0.14	0.06	-0.05	0.31			2003/12/17	0.15	0.06	-0.11	0.31
		2004/11/19	0.35	0.06	0.15	0.49			2004/11/19	0.35	0.06	0.12	0.52
